# Computer simulation study of the penetration of pulsed 30, 60 and 90 GHz radiation into the human ear

**DOI:** 10.1038/s41598-020-58091-7

**Published:** 2020-01-30

**Authors:** Zoltan Vilagosh, Alireza Lajevardipour, Andrew Wood

**Affiliations:** 1 Swinburne University of Technology Melbourne, Melbourne, Australia; 2 Australian Centre for Electromagnetic Bioeffects Research, Melbourne, Australia

**Keywords:** Risk factors, Electrical and electronic engineering

## Abstract

There is increasing interest in applications which use the 30 to 90 GHz frequency range, including automotive radar, 5 G cellular networks and wireless local area links. This study investigated pulsed 30–90 GHz radiation penetration into the human ear canal and tympanic membrane using computational phantoms. Modelling involved 100 ps and 20 ps pulsed excitation at three angles: direct (orthogonal), 30° anterior, and 45° superior to the ear canal. The incident power flux density (PD) estimation was normalised to the International Commission on Non-Ionizing Radiation Protection (1998) standard for general population exposure of 10 Wm^−2^ and occupational exposure of 50 Wm^−2^. The PD, specific absorption rate (SAR) and temperature rise within the tympanic membrane was highly dependent on the incident angle of the radiation and frequency. Using a 30 GHz pulse directed orthogonally into the ear canal, the PD in the tympanic membrane was 0.2% of the original maximal signal intensity. The corresponding PD at 90 GHz was 13.8%. A temperature rise of 0.032° C (+20%, −50%) was noted within the tympanic membrane using the equivalent of an occupational standard exposure at 90 GHz. The central area of the tympanic membrane is exposed in a preferential way and local effects on small regions cannot be excluded. The authors strongly advocate further research into the effects of radiation above 60 GHz on the structures of the ear to assist the process of setting standards.

## Introduction

There is increasing interest in wireless communication systems such as the 5 G mobile networks using the 30 to 90 GHz frequency band^1^. Devices operating in this band use less power per unit of data transmitted than the current frequencies, and the band itself offers data rates in the order of 10 Gigabits per second. Additional applications in the neighbourhood of 30 to 90 GHz include Wireless Local Area Networks (WLAN) operating at 60 GHz^2,3^, millimetre wave radiation heating for material processing^4^, automotive radar at 24–29 GHz and 76–81 GHz^5^, and the Active Denial anti-personnel system at 94 GHz^6^. The deployment of these applications will inevitably have an impact on the environmental exposure of humans.

The absorption coefficient of liquid water increases from about 3500 m^−1^ (35 cm^−1^) at 30 GHz to 7500 m^−1^ (75 cm^−1^) at 90 GHz^7^. It follows that the presence of water in the atmosphere produces significant signal loss, which necessitates denser communication networks with transmitters placed at many angles and elevations. The water content of living soft tissues is in the order of 70–75%, thus most of the energy from any incident radiation is strongly absorbed in the first few millimetres of soft tissue. The study of 30–90 GHz radiation exposure inevitably becomes focused on the skin, the cornea of the eye and the ear canal. The International Commission on Non-Ionizing Radiation Protection (ICNIRP,1998)^8^ standard for exposure to the 10–300 GHz frequency radiation is stated in terms of incident power flux density (PD). The existing PD exposure limits for 10–300 GHz radiation are given as 10 Wm^−2^ for the general public and 50 Wm^−2^ for occupational contact. The PD values translate to an incident orthogonal electric field intensity of 61.4 Vm^−1^ and 137 Vm^−1^ respectively, in air. The draft ICNIRP (2018)^9^ recommendations increase the maximum PD to 20 Wm^−2^ and 100 Wm^−2^ for the general public and occupational exposure respectively. The revised standard is to be released shortly, but the results of this study are easily scalable to any new exposure limits. Given that the human tympanic membrane (ear drum) is exposed to the outside environment through the outer ear canal, there is a need to adequately explore the penetration of 30–90 GHz radiation into the ear. There are no specific ear exposure guidelines in the ICNIRP (1998) standards.

The anatomy of the human ear shows large individual variability^10,11^. The external ear canal is approximately cylindrical. The radius of the adult human canal is 3.0 to 4.5 mm, larger in the vertical dimension than the horizontal and sloping upward and forward from its external opening. The canal becomes wider inferiorly at the tympanic membrane.

The waveguide cut-off frequency for electromagnetic radiation for a cylinder is expressed as *f*_(cutoff)_ = 1.841 C/2π***a***, where *f*_(cutoff)_ is the cutoff frequency below which the cylinder will not function as a waveguide, C is the speed of light in the particular medium and ***a*** is the radius of the cylinder. Given that the radius the ear canal is 3.0–4.5 mm, the expected waveguide cutoff is 18–30 GHz. It follows that the radius of the ear canal does not impede the propagation of the radiation at 30–90 GHz. The absorption and reflection properties of the tissues of the outer ear, the ear canal and the tympanic membrane become important considerations. The diffraction from the structures of the outer ear and the canal entrance is also a significant factor as it modifies or impedes the progress of the radiation. Significant penetration of the radiation into the ear canal at 60 GHz has been demonstrated^12^ and simulations at 300 GHz have shown that 54% of the PD presented to the front of the ear penetrates into the tissues of the tympanic membrane^13^. There are no comparable comprehensive exposure studies for the 30–90 GHz range.

The lining of the outer region of the ear canal resembles normal thin skin. The deep part of the ear canal, on the other hand, has no dermis, a thin 0.01–0.02 mm layer of epidermis and a thicker 0.10 mm layer of stratum corneum (SC) compared with normal skin as the area is not abraded mechanically^14^. In adult humans, the tympanic membrane is approximately 25 mm from the ear canal entrance. The tympanic membrane slopes inward from its superior margin at an angle of 25–30°. The thickness of the tympanic membrane varies with age. The adult tympanic membrane has four layers; two epidermal layers of about 0.02 mm thickness on the outer aspect (consisting of a cornified SC layer and a living basal layer), a fibrous tissue layer of 0.02–0.230 mm (thicker superiorly in the region of the pars flaccida and thinner in the pars tensa), and a 0.02–0.03 mm mucosal lining on the inner aspect of the tympanic membrane^9^. There is a system of epithelial cell migration which is unique to the tympanic membrane and the ear canal which serves to move cells towards the canal entrance^15^, continually clearing debris from the canal.

The tympanic membrane is well supplied with blood, richly innervated and highly sensitive to inflammation and mechanical insult. Tympanic membrane damage may lead to pain or hearing loss. Small changes in the local environment may elicit noticeable symptoms. Heat transfer from the blood flow within the tympanic membrane is supplemented by the diffusion of heat generated by metabolic processes within the brain and the proximity of the carotid artery and jugular vein^16^ and heat loss is aided by loss of heat to the air in the ear canal. The tympanic membrane is thin and is suspended between two air containing cavities of the middle ear and the ear canal, and thus capable of rapid radiative heat loss. The tympanic membrane may be inflamed, increasing the blood flow and temperature or have scarring from past perforations or grommet tube insertions with a reduced blood flow in the scarred area. These features make any thermal effect of a given dose of direct radiation on the tympanic membrane difficult to evaluate. Estimates of temperature rise are, however, possible using the known mass density and thermal properties of the constituent tissues. The function of the tympanic membrane is determined by its structure rather than any specialised tissue components. Any non-thermal effects are likely to be comparable to similar tissues at other sites and need not be studied directly, however, the structures beyond the membrane, such as the vestibular apparatus and cochlea, do contain highly specialised and sensitive tissues, and the level of radiation penetrating to these areas needs particular evaluation.

The study of the human ear is hampered by a lack of a suitable animal model. The usual laboratory animals, such as mice, rats, guinea pigs, rabbits and pigs, have either a disproportionate tortuosity of their ear canal, or have obliquely angled or small tympanic membranes^17^. Given these difficulties, the use of a computational phantom modelling becomes useful for performing the preliminary studies. The aim of the simulations was to investigate the proportion of the radiation that would reach the tympanic membrane at 30, 60, and 90 GHz frequencies in the first instance, and then study the PD, the specific absorption rate (SAR), the temperature rise within the membrane itself and the subsequent propagation of the radiation into the middle ear.

## Results

### The attenuation and distribution of the pulsed signal within the ear canal

**Figure 1:**
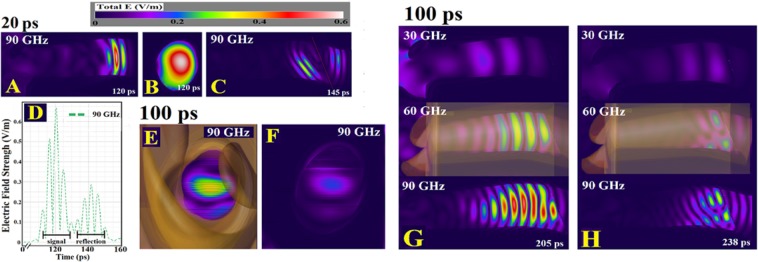
(**A**) The absolute value of the E-field at 90 GHz with the longitudinal planar sensor output, 20 ps orthogonal pulse. (**B**) Planar sensor at 20 mm within the canal showing the increased signal in the central region. (**C**) Longitudinal planar sensor at 145 ps with significant reflection from the surface of the tympanic membrane. (**D**) Time domain variation of the absolute value of the E-field, point sensor 20 mm into the canal. (**E**) Planar sensor placed on the surface of the tympanic membrane (maximal intensity). (**F**) Planar sensor at 0.03 mm in the tympanic membrane. The central region of the tympanic membrane receives the bulk of the incident radiation. (**G**) 100 ps orthogonal pulse sensor output at 205 ps into the simulation showing attenuation of the signal near the edges. (**H**) Planar sensor output at 238 ps with reflection from the surface of the tympanic membrane causing destructive interference.

The diffraction at the outer ear entrance and the reflections from the outer ear structures and within the ear canal change the Gaussian pulses (see Methods) into more complex forms. The 20 ps pulse (at 90 GHz only) shows the progress of the incident and reflected signals more clearly, whilst the 100 ps pulse is superior at demonstrating interference effects within a given pulse. Both the planar sensor at 20 mm and the longitudinal planar sensor show a preferentially exposed central region of the ear canal (Fig. 1A,B). The strong reflection of the signal from the surface of the tympanic membrane is displayed in Fig. 1C. The maximum reflected E-field at the 20 mm point sensor is noted to be 44% of the value for the incoming pulse at the same site (Fig. 1D). For the 90 GHz simulation, the attenuation of the E-field at 20 mm in the canal between 20 ps and 100 ps pulses was 23%, indicating significant destructive interference from reflections in the longer pulse. The reflection at the surface of the tympanic membrane and the consequent reduction of the absorbed signal at 90 GHz is illustrated in Fig. 1(E,F).

The longitudinal planar sensor output for 30, 60 and 90 GHz showing the absolute E-field value with the 100 ps pulse is shown in Fig. 1(G). There is marked attenuation of the signal with decreasing frequency. The complexity of the reflection at the tympanic membrane with the 100 ps pulse using the longitudinal planar sensors is shown in Fig. 1(H). The simulations show a strong interaction between the diffraction of the incident radiation at the structures of the outer ear and the ear canal entrance, leading to a reduction of the incident radiation entering the ear canal. Since diffraction is inversely correlated with frequency, the greatest influence is at 30 GHz. The reflections within the canal and the front surface of the tympanic membrane produced a complex pattern with a central high intensity region.

### The attenuation of the signal with differing angles of excitation

The mean absolute E-field values taken from the six point sensor array 0.03 mm within the tympanic membrane, and the absolute values of the H-field for the direct excitation are presented in Table 1. The mean PD within the tympanic membrane and the maximum value of the PD for the array and the central point sensor are also displayed. The values from the orthogonal, 30° anterior, and 45° superior excitations for the 100 ps pulse at 30, 60 and 90 GHz are shown along with the 20 ps simulation at 90 GHz. The standard deviation (SD) within each dataset is also shown. The paired E-field and H-field values from each point sensor were used to calculate the associated PD.

**Table 1:** The mean absolute electric and magnetic field values.

Electric Field							Magnetic Field		
100 ps	orth.	SD	30° ant.	SD	45° sup.	SD	100 ps	orthogonal	SD
GHz	Vm^−1^		Vm^−1^		Vm^−1^		GHz	mAm^−1^	
30	1.4	0.28	1.32	0.24	1.52	0.29	30	14	3.0
60	4.37	1.09	2.4	0.52	0.76	0.21	60	54	11
90	7.94	2.02	2.33	0.56	0.52	0.04	90	95	2.0
**Electric Field**									
**20 ps**	**orth**.		**30° ant**.		**45° sup**.				
**GHz**	**Vm**^**−1**^	**SD**	**Vm**^**−1**^	**SD**	**Vm**^**−1**^	**SD**			
90	8.75	2.78	2	0.51	0.51	0.045			
**Power Density (PD)**							**Maximum PD**		
**100 ps**	**orth**.		**30° ant**.		**45° sup**.		**100 ps**	**orthogonal**	
**GHz**	**mWm**^**−2**^	**SD**	**mWm**^**−2**^	**SD**	**mWm**^**−2**^	**SD**	**GHz**	**mWm**^**−2**^	
30	20	5.0	18	7.0	24	7.0	30	36	
60	250	11	72	25	77	4.0	60	410	
90	790	35	65	3.0	3.0	0.3	90	1380	
**Power Density (PD)**							**Maximum PD**		
**20 ps**	**orth**.		**30° ant**.		**45° sup**.		**20 ps**	**orthogonal**	
**GHz**	**mWm**^**−2**^	**SD**	**mWm**^**−2**^	**SD**	**mWm**^**−2**^	**SD**	**GHz**	**mWm**^**−2**^	
90	1000	580	49	22	3.1	0.6	90	2070	

E-field, H-field and PD values from the point sensor array within the tympanic membrane, adjusted to an incident signal value of 61.4 Vm^−1^, (equivalent to incident PD of 10 Wm^−2^). The mean E-field and mean PD with standard deviation (SD) with the orthogonal, 30° anterior and 45° superior pulses, with the 100 ps excitation is shown. The H-field values are only for the 100 ps orthogonal excitation. The 20 ps excitation values are shown at 90 GHz only. The maximum PD at the centre of the tympanic membrane with the 100 ps and 20 ps excitations are shown.

In line with the planar sensor data, the central point sensors within the tympanic membrane showed consistently 40–50% higher E and H fields compared with the peripheral sensors. The mean values from the point sensors show a wide dispersion, with standard deviations of the E-field and H-field typically in the order of 30% of the total value. The values were adjusted to an incident signal value of 61.4 Vm^−1^, equivalent to incident PD of 10 Wm^−2^, to bring them in line with the ICNIRP (1998)^8^ maximum exposure recommendations for the general public.

The penetration of the signal into the tympanic membrane at 30 GHz is uniformly low at all angles. There are idiosyncratic, unpredictable variations in the maximum value; in fact the 30 GHz signal has the greatest penetration into the tympanic membrane with the 45° simulation. This effect is explained by the diffraction by structures of the outer ear and the ear canal entrance. At 60 and 90 GHz, there is marked attenuation in the E-field and H-field (and hence fthe PD) at the 30° anterior and 45° superior angles, when compared with the orthogonal excitation. This is explained by the combination of the reduced diffraction at the outer ear and the decreased refractive index with increasing frequency; once the signal enters the ear canal, a greater proportion of the 60 and 90 GHz pulse is absorbed at each interaction with the canal wall.

### The power flux density of the direct signal beyond the tympanic membrane

The PD derived from the sensor array 0.02 mm within the middle ear is shown in Table 2. The value of the PD penetrating to middle ear as the fraction of PD at the array within the tympanic membrane is also presented.

**Table 2:** The mean PD reaching the middle ear.

Orthogonal excitation		% transmitted into	
100 ps	Mean PD	The middle ear	
GHz	mWm^−2^	GHz	%
30	8.3	30	27
60	71	60	18
90	230	90	19

The mean PD for the direct excitations as measured at the of the six point sensors 0.02 mm beyond the tympanic membrane, adjusted for an incident PD of 10 Wm-2. and the fraction of the PD reaching the tympanic membrane array that is transmitted into middle ear.

The data suggest that 60–70% of the radiation reaching the array at 0.03 mm in the tympanic membrane did not enter the middle ear and was either absorbed or was reflected back into the ear canal. Since the absolute value of the PD is trivial at 30 GHz, the transmission has no practical significance. The higher penetration of the 60 GHz and 90 GHz pulses results in approximately 2.3% of the PD incident on the outside of the ear canal being transmitted into the middle ear at 90 GHz.

### SAR and thermal effects on the tympanic membrane

The values for the specific absorption rate (SAR) and temperature rise were based on the mean 100 ps excitation E-field values from Table 1. They are adjusted for both the general public and occupational exposure ICNIRP(1998) recommendations of a maximum PD of 10 Wm^−2^, equivalent incident electric field (E-field) strength in free space is 61.4 Vm^−1^ and are presented in Table 3. The occupational exposure values are a 5x multiple of the values for the general public.

**Table 3:** SAR and thermal studies.

SAR (general public) 61.4 Vm^−1^				Init. Temp. rise method #1 61.4 Vm^−1^			
GHz	orth.	30° ant.	45° sup.	GHz	orth.	30° ant.	45° sup.
	Wkg^−1^	Wkg^−1^	Wkg^−1^			C° s^−1 × ^10^−3^	
30	0.029	0.026	0.034	30	0.019	0.017	0.022
60	0.45	0.14	0.014	60	0.30	0.090	0.0090
90	1.94	0.17	0.0085	90	1.29	0.11	0.0057
**Init. Temp. rise method #2 61.4 Vm**^**−1**^				**Temp. rise after 5** **s (public) 61.4 Vm**^**−1**^			
	**orth**.	**30° ant**.	**45° sup**.		**orth**.	**30° ant**.	**45° sup**.
**GHz**		**°C s**^**−1 × **^**10**^**−3**^		**GHz**		**°C s**^**−1 × **^**10**^**−3**^	
30	0.012	0.011	0.015	30	0.095	0.085	0.11
60	0.27	0.077	0.083	60	1.50	0.45	0.04
90	1.09	0.09	0.0041	90	6.45	0.55	0.028

SAR and temperature rise in the tympanic membrane, calculated using the mean absolute E-field 100 ps excitation values in Table 1. Values are adjusted for the ICNIRP general public PD exposure level of 10 Wm^−2^ (incident E-field of 61.4 Vm^−1^). Initial temperature rise, method #1, uses Eq. (5). The Initial temperature rise, method #2, uses Eq. (7). The total after a simulated 5.0 s exposure, uses Eq. (4).

The thermal impact of the radiation is trivial at 30 GHz and at non-orthogonal angles of excitation for all frequencies. The impact is maximal at occupational exposure levels with the orthogonal excitation at 90 GHz, where the initial temperature rise was 0.00645 °C s^−1^, which corresponds to an estimate of a maximum temperature rise of 0.032 °C (+20%, −50%) for a sustained 5 s exposure at 90 GHz, (with the occupational, orthogonal exposure). Since the tympanic membrane is capable of both radiative heat loss and heat dissipation by way of the blood flow, past simulations have shown that a thermal equilibrium is reached in times shorter than 5.0 s. As the calculations were taken from the maximum value of the SAR with a short pulsed excitation, the thermal impact should be regarded as the theoretical maximum. If the draft ICNIRP(2008)^9^ recommendations are adopted, and the new guidelines increase the recommendations of a maximum PD of 20 Wm^−2^ and 100 Wm^−2^ for the general public and occupational exposure respectively, the thermal impact will still be very limited.

The two methods of estimating initial temperature rise are in good agreement, and given the small contribution by the blood flow and perfusion factors in Eq. (4), the simpler Eq. (5) is a good approximation.

## Discussion

The computational phantom modelling indicates that the proportion of 30 to 90 GHz frequency radiation entering the ear canal and impacting on the tympanic membrane is dependent on the incident angle of the radiation, the signal duration, the location of the region of interest on the tympanic membrane and the radiation frequency. Radiation from indirect angles of incidence penetrate the ear canal at a much lower intensity compared with orthogonal pulses. The variations of the radiation penetration and exposure pattern are explained by the interplay between the diffraction at the outer ear structures, the refractive index and the absorption coefficient of the tissues. Since diffraction reduces with increased frequency, the greatest effect is at 30 GHz, where it is the dominant factor. The tissue absorption coefficient increases and refractive index reduces with increased frequency. The combination of these factors makes the angle of incidence of the radiation more important as the frequency increases.

There is very low penetration of signal into the ear canal at 30 GHz at all angles, which gives reassurance that the rollout of 5 G networks in the 30 GHz region is not likely to have a significant thermal impact on the tympanic membrane. If the draft ICNIRP (2018) recommendations are adopted, and the new guidelines increase the maximum PD to 100 Wm^−2^ for occupational exposures, the impact at 30 GHz will still be negligible. A similar conclusion can be made regarding radiation directed at angles away from the ear canal at all studied frequencies. The only possible concern may be subtle effects with direct orthogonal radiation into the ear canal.

With an orthogonal 100 ps excitation at 90 GHz, the PD 0.03 mm within the tympanic membrane reaches a maximum of 0.79 Wm^−2^, (adjusted for the ICNIRP (1998) general public limit). The SAR for the general public and occupational exposure limits in the same situation reached 3.87 Wkg^−1^ and 19.3 Wkg^−1^ respectively. The calculated maximum temperature rise of 0.032 °C (+20%, −50%) is noted after a 5.0 second exposure at the 90 GHz, using an orthogonal excitation at the occupational limit of 50 Wm^−2^. Although the local SAR is high, the thermal impact of the exposure is negligible because of the rapid heat loss already mentioned.

The central region of the tympanic membrane has a greater exposure to the incident radiation, which raises the possibility of local effects within small regions of the tympanic membrane that may not be evident on studying the tympanic membrane as a whole. Scarring from old trauma or old grommet tube insertion will alter the permittivity and thickness of the effected tympanic membrane regions, producing more uncertainty regarding SAR and thermal impact. In addition, the length and radius of ear canal between adult individuals can vary and this will affect the cutoff frequency and the refraction pattern within the ear canal.

The simplified model used in this simulation is insufficient to make authoritative predictions of the effect of 60 to 90 GHz radiation on the middle ear. As the communications frequencies increase to regions beyond 60 GHz and other applications are deployed, the penetration of radiation into the tympanic membrane and middle ear may become significant, particularly in the case of people with acute or chronic tympanic membrane perforations, where the membrane is not absorbing part of the incident radiation.

The standards are currently undergoing revision, with a view to relaxing the PD levels for skin exposure to 20 Wm^−2^ and 100 Wm^−2^, for the general public and occupational exposures respectively. There is no specific ear canal or tympanic membrane standard for radiation exposure in the ICNIRP (1998) standards for 10–300 GHz.

Given the poor penetration into the ear of the 30 GHz signal, no specific recommendations of standards regarding the ear structures are justified. However, there is a need to explore any likely effects at frequencies of 60 GHz and higher. Whist the tympanic membrane itself does not contain unique tissue components, the structures beyond the membrane, such as the vestibular apparatus and cochlea do contain highly specialised tissue. The effect of exposure may be small and difficult to detect, but it may involve subtle asymmetric heating of the healthy or damaged tympanic membrane, the generation of air convection currents in the middle ear, or a thermally induced fluid movement in the cochlear or the vestibular apparatus. The authors strongly advocate further research into the effects of radiation above 60 GHz on the structures of the ear to assist the process of ongoing standards review.

## Methods

### Model design

**Figure 2:**
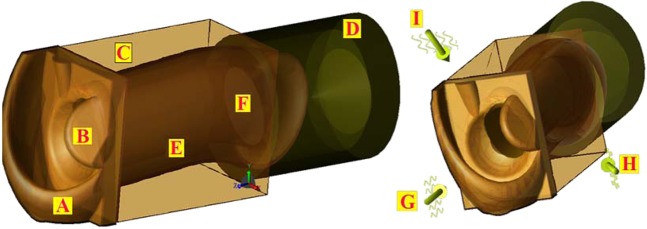
The design of the ear model. (**A**) The antitragus and antihelix. (**B**) The tragus. (**C**) Bone/cartilage padding to prevent lateral encroachment of radiation. (**D**) Rear baffle. (**E**) Ear canal. (**F**) Tympanic membrane. (**G**) Orthogonal angle of excitation. (**H**) 30° Anterior excitation. (**I**) 45° Superior excitation.

The model (Fig. 2) includes the features of the inner region of the pinna (external ear) and the ear canal (3.5 mm radius at its narrowest section horizontally, 4.5 mm radius vertically and 25 mm deep). If the narrowest radius is considered, the waveguide formula (see methods) delivers an estimated cutoff frequency of 25.1 GHz. The tympanic membrane is angled at 25° sloping inward from its superior margin, with a simplified structure of a 0.01 mm stratum corneum, a 0.02 mm epidermal layer a 0.08 mm fibrous tissue layer and an inner mucosal layer of 0.04 mm. The mucosal layer was modelled thicker than the anatomical thickness to account for the wet mucous attached to the lining. A baffle was placed at the rear of the model to diffuse any reflected signal.

Simulations were conducted with an excitation directly into the ear canal as judged by the plane of the outer opening (“orthogonal”), and angles of 30° anterior and 45° superior to the orthogonal. The angled excitations served to explore any protective effect of the outer ear and the canal. The direct and 30° anterior excitations mimic a head high source such as automotive radar impacting on a car passenger, and the 45° superior excitation modelled a pole or ceiling mounted wireless communication transmitter.

**Figure 3:**
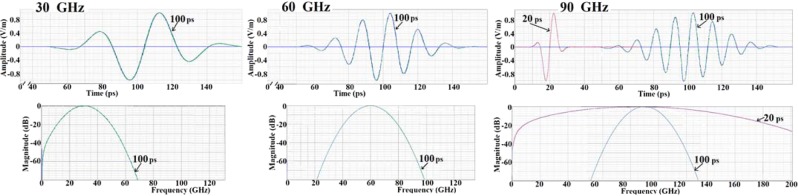
The time domain characteristics and bandwidth of the excitations of the 100 ps duration of 30, 60 and 90 GHz, pulses, and the 90 GHz 20 ps pulse. The excitation amplitude shown is before the adjustment to the notional incident radiation to 61.4 Vm^−1^ and 137 Vm^−1^ for comparison with ICNIRP (1998) standards.

The characteristics of the excitations are presented in Fig. 3. In order to minimize the effects of reflections, far field Gaussian excitation pulses of 100 ps duration were used, modelled separately at 30, 60 and 90 GHz. A 100 ps pulse allows for the passage of 3, 6 and 9 wavelengths at 30, 60 and 90 GHz respectively, at a relatively narrow bandwidth. An additional study of the characteristics of the reflection at the tympanic membrane was undertaken at 90 GHz using a 20 ps pulse (1.8 wavelengths) to separate the incoming and reflected pulses inside the canal. A 20 ps pulse is less than a wavelength period at 30 and 60 GHz and would not produce valid simulations.

The amplitude of the incident excitation signal within the simulation was set at 1.0 Vm^−1^ maximum amplitude. To assess the impact of the ICNIRP recommendations, the electric and the magnetic field (E-field and H-field) results were adjusted to a hypothetical incident radiation of 61.4 Vm^−1^ and 137 Vm^−1^ to correspond with the PD of 10 Wm^−2^ and 50 Wm^−2^, matching the present general public and occupational exposure limits. The simulations did not consider any non-linear responses at differing incident PD intensities, any cumulative effects over time or the effect of heating of the tissues on dielectric values or thermal parameters.

**Figure 4:**
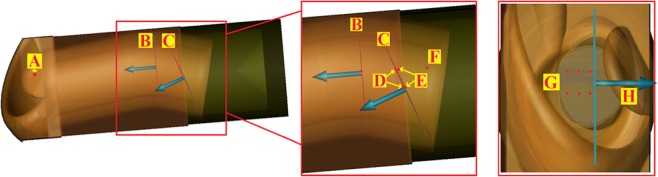
The placement of the sensors registering the absolute value of the E-field (planar and point sensors) and H-field (point sensors only). (**A**) Point sensor at the entrance of the ear canal. (**B**) Planar sensor 20 mm inside the canal. A point senor was placed centrally at the same level. (**C**) Planar sensors at the surface and 0.03 mm inside the tympanic membrane. (**D**) Point sensor array 0.03 mm within the tympanic membrane. (**E**) Point sensor array 0.02 mm within the middle ear. (**G**) The tympanic membrane point sensor array viewed from canal entrance. (**H**) Location of planar sensor placed longitudinally within the ear canal.

The position of the sensors is illustrated in Fig. 4. Point sensors registering the absolute value of the E- and H- fields were placed at the entrance of the ear canal and centrally 20 mm from the canal entry. Point sensors registering the E- and H-fields in an array of 6 were placed 0.03 mm inside the tympanic membrane, at the junction of the epidermis and fibrous tissue. An array of six point sensors was placed 0.03 mm inside the tympanic membrane, at the junction of the epidermis and fibrous tissue. A matching array was placed 0.02 mm past the membrane within the middle ear. Four planar sensors measuring the absolute value of the E-field were placed vertically at 20 mm into the canal, longitudinally bisecting the ear canal, at the surface of the tympanic membrane and at 0.03 mm within the tympanic membrane (at the level of the point sensor array).

Precise computer simulation models require accurate dielectric values for the tissue phantoms. The dielectric values used in the simulation, in the form of the real (***ε***′) and imaginary (***ε***″) part of the complex permittivity at 37 °C, are outlined in Table 4. The dielectric values for living tissues are poorly documented it the 30–90 GHz band; there are some direct *in vivo* skin data^18^ and values derived from porcine tissue^19,20^, however, it was necessary to infer a number of parameters from measurements performed below 20 GHz^21^ and from *in-vivo* rabbit skin data extrapolated from 23 GHz^22^. The fibrous layer in the tympanic membrane is assumed to be equivalent to the sclera and the mucous membrane is set to the vitreous humour values^23^. The dielectric values for the stratum corneum were derived using the Maxwell-Wagner mixing formula^24^ with a literature value for the dry component^23^.

**Table 4:** The real (*ε*′) and imaginary (*ε*″) parts of the complex permittivity at 37 °C.

GHz		Bone and cartilage	Dermis	Epidermis	Stratum corneum	Fibrous tissue	Mucous membrane
30	***ε′***	5.5	19.0	16.0	10.0	18.0	31.0
	***ε″***	1.4	16.0	12.5	6.0	18.5	30.6
60	***ε′***	4.0	10.0	10.0	5.0	10.0	11.5
	***ε″***	1.2	11.0	10.0	4.0	15.0	20.0
90	***ε′***	3.2	6.0	5.5	3.4	7.0	8.0
	***ε″***	1.0	8.0	6.0	3.0	13.0	15.0

The values for the real (ε′) and imaginary (ε″) parts of the complex permittivity at 37 °C for the tissues at used in the simulations. The data are from porcine tissue^19^, and from extrapolated data^21–23^.

Since the bulk of the data for the dielectric properties was acquired at room temperature, the values were adjusted for the tympanic membrane temperature of 37 °C in the manner outlined in previously^25^. The method uses adjustment factors for the real (***ε***′) and imaginary (***ε***″) parts of the tissue complex permittivity to modify the values for data obtained at a different temperature to the one that will be simulated. The adjustment is based on the empirical approximation of changes in the ***ε′*** and ***ε″*** of water^7,26,27^ over the relevant temperature range at the desired frequency rather than theoretical modelling. The detail is presented in the Supplementary material.

The effect of any heating on the dielectric values of the tissues as the simulation progressed was not considered.

The stratum corneum (SC) covers the ear structures and thus is the tissue to be considered when calculating reflections at the air interface. The inner tympanic membrane tissues are most important when considering radiation absorption. The fibrous tissue in the tympanic membrane houses nerve endings and blood vessels, and thus is the most appropriate site considerations of exposure studiesscenarios. The refractive index (***n***) of SC decreases and the absorption coefficient ***(α)*** of the tympanic membrane fibrous tissue increases as the radiation frequency increases. The values of ***n*** for the SC are 3.60, 2.39 and 1.99 for 30, 60 and 90 GHz respectively. The combination of the changes in the ***n*** of the SC and the ***α*** of the fibrous tissue provide for a reduced penetration into the ear canal for radiation directed from non-orthogonal sources as the frequency increases on one hand, and a reduced reflection and greater absorption within the tympanic membrane as the frequency increases on the other.

The ear canal entrance and the outer ear structures are sites for diffraction. The distance to the first order minimum (Y) in a diffraction pattern is given by Y ≈ λD_**tm**_/2R_o_, where λ, D_tm_ and R_o_ are the wavelength of the radiation, the distance at which the pattern is assessed at and the radius of the aperture respectively. With the lesser dimension of the ear canal entrance (R_o_) of 3.5 mm, the distance to the tympanic membrane (D_tm_) at ~25 mm, the first order minimum (Y) at the tympanic membrane has a width of 35 mm at 30 GHz, 17.9 mm at 60 GHz and 11.7 mm at 90 GHz. The ear canal itself acts as thick aperture, particularly if the radiation directed at angles in the order of 30°, thus the resultant the diffraction pattern is complex^28^.

The simulation was performed using variable geometry, employing a finite difference time domain (FDTD) solver (XFdtd Bio-Pro, version 7.6.0.5.r48456, Remcom, State College, PA). The maximum and minimum Yee cell dimensions in terms of the wavelength (λ) in air, model size, timestep size and total duration of the simulations are shown in Table 5.

**Table 5:** Yee cell dimensions an time step size.

GHz	λ in air (mm)	min. cell dimension	max. cell dimension	Problem space cells (x, y, z)	Time step ps	Total steps 20 ps pulse	Total steps 100 ps pulse
30	10.0	0.0016λ	0.015λ	405, 475, 620	0.0281		14000
60	5.0	0.0016λ	0.029λ	567, 630, 905	0.0150		35000
90	3.3	0.0015λ	0.029λ	690, 721, 1057	0.0102	22000	35000

Yee cell dimensions, the model size, timestep and total duration of the simulations are outlines. The x, y, z dimensions are as shown in Fig. 1. λ refers to the wavelength in air.

The variation in cell size was necessitated by the proportions of the model, the minimum cell sizes were reserved for the tympanic membrane and the SC of the ear canal lining, with larger sizes used for the less important absorptive padding. The variation in the duration of the simulations was necessitated by the length of time the excitations took to clear the tympanic membrane. Separate simulations were performed for each frequency.

The PD within the tympanic membrane was calculated using the equation:

1 $${\rm{PD}}({{\rm{Wm}}}^{-2})={\rm{E}}({{\rm{Vm}}}^{-1})\,\times \,{\rm{H}}({{\rm{Am}}}^{-1})$$

where E and H are the electric field and magnetic field intensities respectively. The PD within both the air filled ear canal and the air filled middle ear was calculated using the PD = E^2^ /377, where 377 is the impedance of free space (in Ω). The data for the individual sensors is presented in the supplementary material. The SAR was calculated using the equation:

2 $${\rm{SAR}}={\sigma }_{t}{{\rm{E}}}^{2}/2{\rho }_{t},$$

where ***σ***_***t***_, E and ***ρ***_***t***_ are the electrical conductivity, the maximum intensity of the electric field and the mass density of the tissue respectively. Here we assume that the maximum electric field achieved in response to a pulse would be the same as that in response to a continuous incident sine wave of 1.0 Vm-1. ***σ*** can be calculated from the imaginary part of permittivity (***ε″***) using the equation:

3 $$\sigma ={\varepsilon }_{0}\varepsilon ^{\prime\prime} 2\pi f$$

where ***ε***_***0***_ is the permittivity of free space (8.85 × 10^−12^ m^−3^ kg^−1^ s^4^ A^2^), and ***f*** is the frequency of the radiation. The equation yielded a ***σ***_***t***_ of 30.9 Sm^−1^ (30 GHz), 50.1 Sm^−1^ (60 GHz) and 65.1 Sm^−1^ (90 GHz) for the tympanic membrane fibrous tissue. For thermal calculations involving longer periods, it is necessary to use the bioheat equation^29^ in a modified from:

4 $${\Delta T}_{t}=\frac{{k}_{t}{\nabla }^{2}{{\rm{T}}}_{t}+{\rho }_{t}SAR-{\rho }_{b}{c}_{b}\,BP({{\rm{T}}}_{t}-{{\rm{T}}}_{b})}{{\rho }_{t}{c}_{t}}\Delta t$$

where ΔT, is the initial temperature rise (°C s^−1^), ***c***_*t*_ is the heat capacity of the tissue and Δt is an arbitrarily small passage of time, and ***k***_***t***_, T_***t***_ T_***b***_ is the thermal conductivity, and the tissue and blood temperature respectively. ***ρ***_*b*_ ***c***_*b*_ and BP are the blood density, heat capacity of blood and blood perfusion rate in the tissues respectively. If the difference between the tissue temperature and blood temperature is small, the (T_*t*_ − T_*b*_) term is small. Given the blood flow term in SI units is also small, the entire blood flow term can be neglected for short periods of time. If the initial temperature rise is small, the term ***k***_*t*_∇^2^T_*t*_ will also be insignificant. The initial temperature rise, can then be approximated by using the equation quoted in the draft ICNIRP paper^9^:

5 $$\Delta T=({\rm{SAR}}/{c}_{t})\Delta t,$$

An alternate method of calculating the temperature rise at the surface of the skin given by Alexeev and Ziskin^30^**:**

6 $$\Delta T=\frac{2(1-R)I\,}{{{\boldsymbol{\delta }}}_{{\boldsymbol{t}}}{{\boldsymbol{\rho }}}_{{\boldsymbol{t}}}{{\boldsymbol{c}}}_{{\boldsymbol{t}}}}{e}^{-2z/\delta }\Delta t$$

where *R*, I, ***δ***_***t***_ and *z* are the reflected radiation, total incident PD, penetration depth and the depth of tissue under investigation respectively. The numerator in the fraction, 2(1-R)I, is an estimate of the PD at a given depth within the tissue, with an assumption of a uniform tissue ***n*** = 2. Given that the simulation gives a more direct PD estimate for the tissues, the equation reduces to:

7 $$\Delta T=\frac{{\rm{PD}}{e}^{-2z/\delta }}{{{\boldsymbol{\delta }}}_{{\boldsymbol{t}}}{{\boldsymbol{\rho }}}_{{\boldsymbol{t}}}{{\boldsymbol{c}}}_{{\boldsymbol{t}}}}\Delta t$$

The estimation with metabolic heat can be neglected as heat production in resting humans is relatively low^28^. The values for ***ρ***_***t***_, ***c***_*t*_, ***k***_***t***_, and BF are given in Table 6.

**Table 6:** Tissue parameter values for thermal studies.

	Tissue Density *ρ*_*t*_ kgm^−3^	Heat Capacity *c*_*t*_ Jkg^−1^ K^−1^	Thermal Conductivity *k*_*t*_ Wm^−1^ °C^−1^	Blood Flow BF mlkg^−1^min^−1^ m^3^kg^−1^s^−1^	
Stratum Corneum	1300	1800	0.4	0	0
Epidermis	1060	3600	0.6	0	0
Fibrous tissue	1151	3000	0.6	3.8	6.3 ×10^−8^
Mucous membrane	1125	3150	0.5	12.0	2.0 ×10^−7^
Blood	1057	3600	0.52		
Air 35°/37 °C	1.13	1005	0.0026		

The values for the tissue density and thermal properties. Blood flow is given in both the traditional mlkg^−1^ min^−1^ units and SI units (m^3^kg^−1^s^−1^). The blood flow values need to be multiplied by ρt to provide consistent units in Eq. (4).

The fibrous tissue in the tympanic membrane, was set as equivalent to the values for sclera^29^, and the Stratum Corneum values^31^, with other tissue and air parameters are based on the IT’IS Database^32^. The initial temperature of both the interior of the tympanic membrane and blood temperature was set at 37.0 °C. The outer surface of the tympanic membrane is 0.3–0.5 C° cooler than core temperature but this can vary considerably^33^ even by simple procedures as covering the ear canal entrance. There are also two air/tissue boundaries to consider; the tympanic membrane/outer ear canal boundary on one side and the tympanic membrane/middle ear boundary on the other. Additional heat is also generated nearby by metabolic processes within the brain and heat from the carotid artery and jugular vein^16^. These variables make the estimation of convection and radiant heat exchange at the surfaces of the tympanic membrane highly speculative. The thermal impact of radiation on the tympanic membrane was, thus, confined to applying Eqs. (4, 5 and 7). Equation (4) was calculated with a timestep of 0.1 s over a total of 5.0 s, as past simulations have shown that a thermal equilibrium within thin tissues is reached in times shorter than 5.0 s.

The contributions to overall SD have been estimated as follows: ***ε′*** and ***ε″*** (from literature values): 8%; adjustment in ***ε′*** and ***ε″*** due to temperature changes: 2%. Simulations with +/− 10% variation in the ***ε′*** and ***ε″*** at 90 GHz (100 ps, orthogonal pulse) yielded changes in the computed mean maximum E-field of +5.5%, −22.1%. Given that the estimation of SAR and thermal changes depend on the square of the value of the E-field, the estimates for the errors in SAR become +10.7% and −39.3%. Errors in other inputs include tissue thermal properties (literature values) of 5% and FDTD meshing errors of 3%. The total SD of temperature rise estimates is thus in the order of +20%, −50%. In addition, the length of ear canal between adult individuals can vary by 15%^11^, and the radius of the ear canal by as much as 50%^11^. The ear canal dimensions will affect the canal cutoff frequency and the refraction pattern within the ear canal.

## Supplementary information

Supplementary information.

## Data Availability

Added graphics, a fuller explanation of the rationale for using intensities adjusted to the International Commission on Non-Ionizing Radiation Protection (ICNIRP, 1998) and additional data has been presented as supplementary material.
