# Supplementary material for: Computer simulation study of the penetration of pulsed 30, 60 and 90 GHz radiation into the human ear
Source: Sci Rep. 2020 Jan 30;10:1479. doi: 10.1038/s41598-020-58091-7 (PMC6992669; doi:10.1038/s41598-020-58091-7)
Supplement: Supplementary file 1 — Supplementary information. [file 41598_2020_58091_MOESM1_ESM.docx]

**Supplementary material**

**Computer simulation study of the penetration of pulsed 30, 60 and 90 GHz radiation into the human ear**

Zoltan Vilagosh^[[1]](#footnote-1),^^[[2]](#footnote-2)^ *, Alireza Lajevardipour^1,2^, Andrew Wood^1,2,^

1. **Additional views of the ear model**

Additional views of the computational ear model are presented to assist the reader.


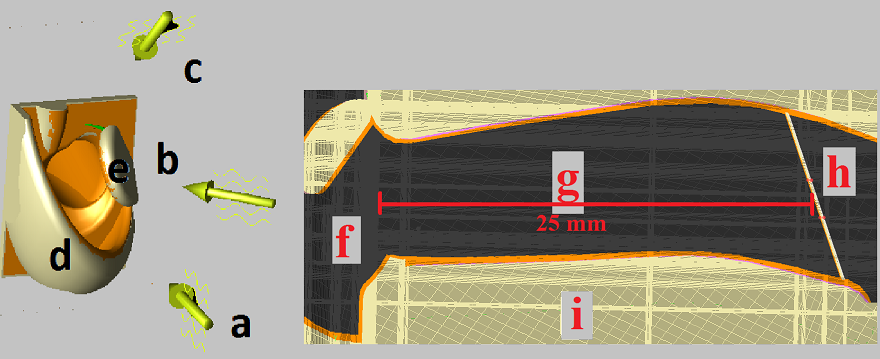


**Figure 1:** Further views of the ear model from behind the ear and a cross section. Excitation directions: **(a)** Orthogonal excitation direction, **(b)** 30^0^ anterior, **(c)** 45^0^ superior. **(d)** The antitragus and antihelix. **(e)** Tragus. **(f)** The ear canal entrance. **(g)** The ear canal. **(h)** The placement of the point sensors registering the absolute value of the E-field and H-field in the tympanic membrane

1. **Detail of the E-field, H-field and Power Flux Density (PD) data from the point sensors in the tympanic membrane and the E-field and PD in the middle ear.**

The point sensors in the tympanic membrane were separated by 0.5 mm laterally and 1.0 mm vertically. As the tympanic membrane sloped 25^0^ from the vertical, the actual vertical separation of the sensors was thus 1.0/ cos(25^0^) ~ 1.1 mm.


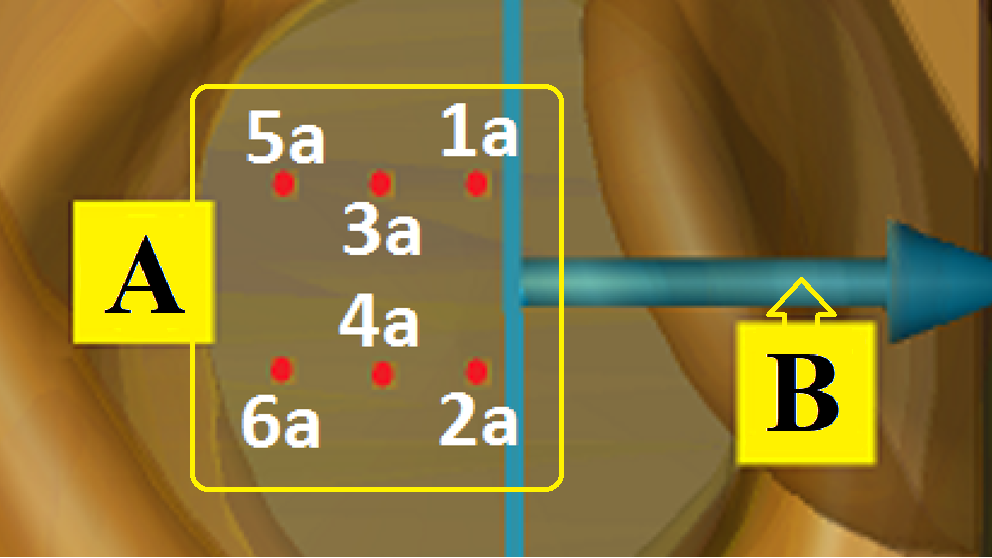


**Figure 2:** **(A)** Detailed nomenclature of the placement of the point sensors, 0.03 mm in the tympanic membrane, at the stratum basale/fibrous tissue boundary. Sensors *1b* to *6b* are in a matching placement, 0.02 mm into the middle ear, behind the *1a* to *6a* array. **(B)** Detail of the placement of the longitudinal planar sensor within the ear canal**.**

1. **Rationale for adjusting the excitation to 61.4 Vm^-1^ and 137Vm^-^**^1^

As noted in the main article, the calculations are adjusted for the incident power density (PD) of 10 Wm^-2^, in line with the International Commission on Non-Ionizing Radiation Protection (ICNIRP) 1998 standard for general public exposure to 10-300 GHz frequency radiation.

The equation to calculate PD (Wm^-2^) in empty space is PD=E^2^ /377, where E is the electric field (Vm^-1^) and 377 (Ω) is the impedance of empty space. Hence, for the incident PD the air containing outer ear canal and middle ear:

$E=\sqrt{377 PD}$ (1)

Thus, to achieve an incident surface PD in empty space of 10 Wm^-2^, the (ICNIRP) 1998 standard for the general public:

$E=\sqrt{377 x 10}=61.4$ (2)

If follows that a 61.4 Vm^-1^ excitation needs to be applied. A similar rationale can be followed for the ICNIRP (1998) standard for occupational exposure of 50Wm^-2^, yielding an excitation intensity of 137 Vm^-1^. The equation for the PD if the medium is not empty space can be expressed in two ways:

$PD=nE^{2} /377$ (3)

where ***n*** is the refractive index (i.e. the PD increases in a material with a higher refractive index). Alternatively, the equation can use the direct measurements of the electric and magnetic fields:

$PD=E.H$ (4)

where the *H* is the magnetic field (Am^-1^). This was the form of the formula used for estimating DP in the tissues from direct measurement of both *E* and *H*.

1. **The data from the sensor arrays**

The data from the sensor arrays is presented in tables 1 to 9. The presents the individual sensor outputs of the simulations at 30, 60 and 90 GHz, at orthogonal, 30^0^ anterior and 45^0^ superior excitations of 100 ps. Sensors *1a* to *6a* refer to the array as described in Fig. 2 of the supplementary material.

| **Table 1: simulation at 30 GHz, orthogonal, 100 ps duration** | | | | | | | |  |
| --- | --- | --- | --- | --- | --- | --- | --- | --- |
|  |  | **Electric field Magnetic field** | | | | | |  |
|  |  | **mV^-1^** | **mVm^-1^** | **mAm^-1^** | **mAm^-1^** |  |  | |
|  | **sensor** |  | **x 61.4** |  | **x 61.4** |  |  |  |
|  | *1a* | 31 | 1903 | 0.22 | 14 |  |  |  |
|  | *2a* | 23 | 1412 | 0.28 | 17 |  |  |  |
|  | *3a* | 26 | 1596 | 0.20 | 12 |  |  |  |
|  | *4a* | 18 | 1105 | 0.31 | 19 |  |  |  |
|  | *5a* | 21 | 1289 | 0.20 | 12 |  |  |  |
|  | *6a* | 18 | 1105 | 0.18 | 11 |  |  |  |
|  |  | **MEAN** | **1402** |  | **14** |  |  |  |
|  |  | **SD** | **282** |  | **2.9** |  |  |  |

| **Table 2: simulation at 30 GHz, 30^0^ anterior, 100 ps duration** | | | | | | | |  |
| --- | --- | --- | --- | --- | --- | --- | --- | --- |
|  |  | **Electric field Magnetic field** | | | | | |  |
|  |  | **mV^-1^** | **mVm^-1^** | **mAm^-1^** | **mAm^-1^** |  |  | |
|  | **sensor** |  | **x 61.4** |  | **x 61.4** |  |  |  |
|  | *1a* | 28 | 1719 | 0.28 | 17 |  |  |  |
|  | *2a* | 25 | 1535 | 0.25 | 15 |  |  |  |
|  | *3a* | 20 | 1228 | 0.19 | 12 |  |  |  |
|  | *4a* | 21 | 1289 | 0.20 | 12 |  |  |  |
|  | *5a* | 18 | 1105 | 0.17 | 10 |  |  |  |
|  | *6a* | 17 | 1043 | 0.17 | 10 |  |  |  |
|  |  | **MEAN** | **1.32** |  | **13** |  |  |  |
|  |  | **SD** | **0.24** |  | **2.5** |  |  |  |

| **Table 3: simulation at 30 GHz, 45^0^ superior 100 ps duration** | | | | | | | |  |
| --- | --- | --- | --- | --- | --- | --- | --- | --- |
|  |  | **Electric field Magnetic field** | | | | | |  |
|  |  | **mV^-1^** | **mVm^-1^** | **mAm^-1^** | **mAm^-1^** |  |  | |
|  | **sensor** |  | **x 61.4** |  | **x 61.4** |  |  |  |
|  | *1a* | 31 | 1903 | 0.28 | 17 |  |  |  |
|  | *2a* | 28 | 1719 | 0.31 | 19 |  |  |  |
|  | *3a* | 26 | 1596 | 0.26 | 16 |  |  |  |
|  | *4a* | 27 | 1658 | 0.16 | 9.8 |  |  |  |
|  | *5a* | 18 | 1105 | 0.26 | 16 |  |  |  |
|  | *6a* | 19 | 1167 | 0.28 | 17 |  |  |  |
|  |  | **MEAN** | **1525** |  | **16** |  |  |  |
|  |  | **SD** | **291** |  | **2.9** |  |  |  |

| **Table 4: simulation at 60 GHz, orthogonal, 100 ps duration** | | | | | | | |  |
| --- | --- | --- | --- | --- | --- | --- | --- | --- |
|  |  | **Electric field Magnetic field** | | | | | |  |
|  |  | **mV^-1^** | **mVm^-1^** | **mAm^-1^** | **mAm^-1^** |  |  | |
|  | **sensor** |  | **x 61.4** |  | **x 61.4** |  |  |  |
|  | *1a* | 100 | 6140 | 1.08 | 66.3 |  |  |  |
|  | *2a* | 88 | 5403 | 1.10 | 67.5 |  |  |  |
|  | *3a* | 70 | 4298 | 0.93 | 57.1 |  |  |  |
|  | *4a* | 61 | 3745 | 0.86 | 52.8 |  |  |  |
|  | *5a* | 60 | 3684 | 0.70 | 43.0 |  |  |  |
|  | *6a* | 48 | 2947 | 0.65 | 39.9 |  |  |  |
|  |  | **MEAN** | **4370** |  | **54.4** |  |  |  |
|  |  | **SD** | **1087** |  | **10.6** |  |  |  |

| **Table 5: simulation at 60 GHz, 30^0^ anterior, 100 ps duration** | | | | | | | |  |
| --- | --- | --- | --- | --- | --- | --- | --- | --- |
|  |  | **Electric field Magnetic field** | | | | | |  |
|  |  | **mV^-1^** | **mVm^-1^** | **mAm^-1^** | **mAm^-1^** |  | | |
|  | **sensor** |  | **x 61.4** |  | **x 61.4** |  |  |  |
|  | *1a* | 52 | 3193 | 0.56 | 34 |  |  |  |
|  | *2a* | 47 | 2886 | 0.57 | 35 |  |  |  |
|  | *3a* | 38 | 2333 | 0.47 | 29 |  |  |  |
|  | *4a* | 37 | 2272 | 0.46 | 28 |  |  |  |
|  | *5a* | 31 | 1903 | 0.40 | 25 |  |  |  |
|  | *6a* | 28 | 1719 | 0.41 | 25 |  |  |  |
|  |  | **MEAN** | **2384** |  | **29** |  |  |  |
|  |  | **SD** | **515** |  | **4.1** |  |  |  |

| **Table 6: simulation at 60 GHz, 45^0^ superior** | | | | | | | |  |
| --- | --- | --- | --- | --- | --- | --- | --- | --- |
|  |  | **Electric field Magnetic field** | | | | | |  |
|  |  | **mV^-1^** | **mVm^-1^** | **mAm^-1^** | **mAm^-1^** |  | | |
|  | **sensor** |  | **x 61.4** |  | **x 61.4** |  |  |  |
|  | *1a* | 18.0 | 1105 | 0.22 | 14 |  |  |  |
|  | *2a* | 15.5 | 952 | 0.19 | 11 |  |  |  |
|  | *3a* | 10.5 | 645 | 0.14 | 8.6 |  |  |  |
|  | *4a* | 12.5 | 768 | 0.15 | 9.0 |  |  |  |
|  | *5a* | 9.5 | 583 | 0.13 | 7.7 |  |  |  |
|  | *6a* | 8.0 | 491 | 0.11 | 6.8 |  |  |  |
|  |  | **MEAN** | **757** |  | **9.5** |  |  |  |
|  |  | **SD** | **213** |  | **2.3** |  |  |  |

| **Table 7, simulation at 90 GHz, orthogonal, 100 ps duration** | | | | | | | |  |
| --- | --- | --- | --- | --- | --- | --- | --- | --- |
|  |  | **Electric field Magnetic field** | | | | | |  |
|  |  | **mV^-1^** | **mVm^-1^** | **mAm^-1^** | **mAm^-1^** |  | | |
|  | **sensor** |  | **x 61.4** |  | **x 61.4** |  |  |  |
|  | *1a* | 183 | 11236 | 2.00 | 123 |  |  |  |
|  | *2a* | 160 | 9824 | 1.8 | 111 |  |  |  |
|  | *3a* | 127 | 7798 | 1.7 | 101 |  |  |  |
|  | *4a* | 110 | 6754 | 1.5 | 92 |  |  |  |
|  | *5a* | 111 | 6815 | 1.2 | 74 |  |  |  |
|  | *6a* | 85 | 5219 | 1.1 | 68 |  |  |  |
|  |  | **MEAN** | **7941** |  | **95** |  |  |  |
|  |  | **SD** | **2021** |  | **19** |  |  |  |

| **Table 8: simulation at 90 GHz, 30^0^ anterior, 100 ps duration** | | | | | | | |  |
| --- | --- | --- | --- | --- | --- | --- | --- | --- |
|  |  | **Electric field Magnetic field** | | | | | |  |
|  |  | **mV^-1^** | **mVm^-1^** | **mAm^-1^** | **mAm^-1^** |  | | |
|  | **sensor** |  | **x 61.4** |  | **x 61.4** |  |  |  |
|  | *1a* | 52 | 3193 | 0.54 | 33 |  |  |  |
|  | *2a* | 48 | 2947 | 0.54 | 33 |  |  |  |
|  | *3a* | 37 | 2272 | 0.50 | 31 |  |  |  |
|  | *4a* | 32 | 1965 | 0.28 | 17 |  |  |  |
|  | *5a* | 33 | 2026 | 0.46 | 28 |  |  |  |
|  | *6a* | 26 | 1596 | 0.26 | 16 |  |  |  |
|  |  | **MEAN** | **2333** |  | **26** |  |  |  |
|  |  | **SD** | **562** |  | **7.2** |  |  |  |

| **Table 9: simulation at 90 GHz, 45^0^ superior 100 ps duration** | | | | | | | |  |
| --- | --- | --- | --- | --- | --- | --- | --- | --- |
|  |  | **Electric field Magnetic field** | | | | | |  |
|  |  | **mV^-1^** | **mVm^-1^** | **mAm^-1^** | **mAm^-1^** |  | | |
|  | **sensor** |  | **x 61.4** |  | **x 61.4** |  |  |  |
|  | *1a* | 9.5 | 583 | 0.101 | 6.2 |  |  |  |
|  | *2a* | 8.7 | 534 | 0.097 | 6.0 |  |  |  |
|  | *3a* | 8.7 | 534 | 0.093 | 5.7 |  |  |  |
|  | *4a* | 8.0 | 491 | 0.085 | 5.2 |  |  |  |
|  | *5a* | 8.3 | 510 | 0.089 | 5.5 |  |  |  |
|  | *6a* | 7.7 | 473 | 0.103 | 6.3 |  |  |  |
|  |  | **MEAN** | **521** |  | **5.8** |  |  |  |
|  |  | **SD** | **36** |  | **0.39** |  |  |  |

1. **Temperature adjustments for the tissues.**

The theoretical mathematical modelling of water interactions in the THz range uses resonant (Kramers–Kronig or Lorentz) and non-resonant (Debye) absorption models, with an added Lorenz factor**^1,2^**. The method used to model ***ε**** generally takes the form of the double Debye formula, with or without a Lorenz term. The Lorenz terms become significant for frequencies above 500 GHz**^1^**^,^**^3,4^** and become the dominant factor above 2.0 THz. The equation for a given frequency (***f***) and temperature (*T* ) is expressed as:

${\boldsymbol{\varepsilon}^{\boldsymbol{*}}}_{f}\left( T \right)={\boldsymbol{\varepsilon}^{\boldsymbol{*}}}_{\infty}\left( T \right)+\left\{ \sum_{i=1}^{2} \frac{\Delta\varepsilon_{i}\left( T \right)}{1+\left( j\frac{f}{ϒ_{a(t)}} \right)^{1-\eta}} \right\}-j\frac{\sigma_{s(T)}}{2\pi{f\varepsilon}_{0}}$

(5)

where:

∆***ε*** _(_$i$_=_1_)_ _=_ ***ε***_s_ ***_–_ ε***_(_$i$_=_1_);_  ∆***ε***_(_$i$_=_2_))_ **_=_ *ε***_(_$i$_=_1_)_ ***_–_ ε***_∞_*_;_*  (6)

where ***ε***_(_$i$_=_1_)_ ***ε***_s_ and ***ε***_∞_**_;_** are the characteristic, static, and the high frequency limit values of ***ε^*^***, ***ε****_0_* is the permittivity of free space and ***σ***_s_ is the DC conductivity of water. The *ϒ*_a(_*_t_*_)_ terms in the denominator are the Debye relaxation frequencies at a particular temperature (*T* ). At 37^0^ C, the values given by Lajevardipour**^4^** are *ϒ*_1_ = 25.1 GHz and ϒ_2_= 157 GHz. For biological materials, a Cole–Cole spread factor (1- *η*) gives a better fit for experimental data; for water and other simple fluids, *η* approaches zero**^4^**.

Equations (5) and (6) bring in 6 variables which are only defined experimentally. Alternate forms of the expression:

. (7)

$$\left( j\frac{f}{ϒ_{i(t)}} \right)^{1-\eta}$$

within equation (5) use the angular velocity (ω) and the relaxation constants ***τ_1_*** and ***τ_2_*** to give the equivalent expressions for *i* = 1 and *i* = 2:

$j$ωτ_1_ and $j$ωτ_2_  (8)

respectively, without the Cole–Cole spread factor. Given that various authors give dissimilar values for the 6 variables and given the alternate forms of the equation, choosing the correct set of variables is a non-trivial task. A sample of values given for ***τ_1_*** and ***τ_2_* _._**by various authors is presented in Table (10):

| **Table 10: variation of *τ_1_* and *τ_2_*  given by various authors.** | | | | |  |
| --- | --- | --- | --- | --- | --- |
| **Temperature** | **τ_1_** | **τ_2_** |  | **Reference** |  |
|  | **picoseconds** | **femtoseconds** |  |  |  |
| **"room"** | 8.40 | 100 |  | Reid.^5^ | |
| **"room"** | 8.30 | 182 |  | Koeberg ^6^ | |
| **"room"** | 8.24 | 180 |  | Kindt & Schmuttenmaer ^7^ | |
| **19^0^ C** | 8.50 | 170 |  | Rønne ^8^ | |
| **none** | 10.60 | 180 |  | Pickwell ^9^ | |
| **22^0^ C** | 8.00 | 180 |  | Fitzgerald ^10^ | |
| **25^0^ C** | 9.30 | 274 |  | Lee ^11^ | |

The theoretical calculations are, paradoxically, dependent on experimentally derived values. Given the large differences in the values given by various authors for the variables, as shown by the spread of the values for ***τ_1_*** and ***τ_2,_*** the decision was made to use a more direct method.

The method outlined by Vilagosh et al. uses adjustment factors for ***ε'*** and ***ε"*** to modify the tissue values based on an empirical approximation of changes in the ***ε'*** and ***ε"*** of water over the relevant temperature range at the desired frequency. The method employs the data from direct measurements given in literature, rather than theoretical modelling. The ***ε'*** and ***ε"*** of water in the range 30 to 90 GHz varies considerably, thus, unlike the THz range**^12^**, separate adjustment factors need to be used for each frequency.

The estimates rely on data from Liebe et al.^1^, Ellison et al.^13^ and Buchner et al.^14^.

**For 30 GHz:**

Δ***ε'*** = 0.584t+12.03

Δ***ε"*** = 0.0077t^2^+0.772t+22.60

**For 60 GHz:**

Δ***ε'*** = 0.224t+6.01

Δ***ε"*** = 0.369t+12.06

**For 90 GHz:**

Δ***ε'*** = 0.003t^2^-0.018x+6.9

Δ***ε"*** = 0.266t+8.7

Where t is the temperature in degrees C.

**References for Supplementary Material.**

1. Liebe, H. J., Hufford, G. A., & Manabe, T. A model for the complex permittivity of water at frequencies below 1 THz. *International Journal of Infrared and Millimeter Waves,* **12,** 659-675, (1991).
2. Cherkasova, O., Nazarov, M., & Shkurinov, A. Properties of aqueous solutions in THz frequency range*. Journal of Physics, Conference Series* **793**(1), 012005, (2017).
3. Shubitidze, F., & Österberg, U. Phenomenological model to fit complex permittivity data of water from radio to optical frequencies. *Physical Review E,* **75**(4), 046608, (2007).
4. Lajevardipour, A., Wood, A.W., McIntosh, R.L. & Iskra, S., Estimation of dielectric values for tissue water in the Terahertz range. *Bioelectromagnetics*, **37**(8),563-567. (2016).
5. Reid, C.B., Spectroscopic Methods For Medical Diagnosis At Terahertz Wavelengths, Doctoral dissertation, University College London, (2009)
6. Koeberg, M., Wu, C. C., Kim, D., & Bonn, M. THz dielectric relaxation of ionic liquid, water mixtures. *Chemical physics letters,* **439**(1),60-64. (2007).
7. Kindt J.T., & Schmuttenmaer C.A. Far-Infrared Dielectric Properties of Polar Liquids Probed by Femtosecond Terahertz Pulse Spectroscopy, *Journal of Physical Chemistry.* **100**,10373-10379,(1996)
8. Rønne , C., Thrane, L., Åstrand, P. O., Wallqvist, A., Mikkelsen, K. V., & Keiding, S. R. R. Investigation of the temperature dependence of dielectric relaxation in liquid water by THz reflection spectroscopy and molecular dynamics simulation. *The Journal of Chemical Physics,* **107**(14),5319-5331, (1997).
9. Pickwell, E., Cole, B. E., Fitzgerald, A. J., Pepper, M., & Wallace, V. P. (2004). In vivo study of human skin using pulsed terahertz radiation. *Physics in Medicine and Biology*, **49**(9),1595.
10. Fitzgerald, A. J., Pickwell-MacPherson, E., & Wallace, V. P. Use of finite difference time domain simulations and Debye theory for modelling the terahertz reflection response of normal and tumour breast tissue. *PloS one,* **9**(7), e99291, (2014).
11. Lee, S., Kang, H., Do, Y., Lee, G., Kim, J. & Han, H., High-precision THz Dielectric Spectroscopy of Tris-HCl Buffer. *Journal of the Optical Society of Korea*, **20**(3),431-434. (2016).
12. Lewis, R. A. (2014) A review of terahertz sources. Journal of Physics D, Applied Physics 47(37), 374001.
13. Vilagosh, Z., Lajevardipour, A., & Wood, A. An empirical formula for temperature adjustment of complex permittivity of human skin in the terahertz frequencies. *Bioelectromagnetics*, **40**, 74-79. (2019).
14. Ellison, W. J., Lamkaouchi, K., & Moreau, J. M. Water: a dielectric reference. *Journal of Molecular Liquids,* **68**(2-3), 171-279. (1996).
15. Buchner, R., Barthel, J., & Stauber, J. The dielectric relaxation of water between 0 C and 35 C. *Chemical Physics Letters,* **306,** 57-63. (1999).

1. Swinburne University of Technology Melbourne, Australia [↑](#footnote-ref-1)
2. Australian Centre for Electromagnetic Bioeffects Research, Australia

   * Correspondence: zvilagosh@swin.edu.au [↑](#footnote-ref-2)
